# Lost in translation

**DOI:** 10.12688/f1000research.15020.2

**Published:** 2019-01-02

**Authors:** Parashkev Nachev, Geraint Rees, Richard Frackowiak

**Affiliations:** 1 Institute of Neurology, University College London, London, WC1N 3BG, UK; 2 Institute of Cognitive Neuroscience, University College London, London, WC1N 3AR, UK; 3 Faculty of Life Sciences, University College London, London, WC1E 6BT, UK; 4 Wellcome Trust Centre for Neuroimaging, University College London, London, WC1N 3BG, UK; 5 Ecole Polytechnique Federale de Lausanne - Faculty of Life Sciences, Blue Brain Project, Geneva, Switzerland

**Keywords:** Translation, high-dimensional inference, causality, neuroimaging, cognitive neuroscience, machine learning.

## Abstract

Translation in cognitive neuroscience remains beyond the horizon, brought no closer by supposed major advances in our understanding of the brain. Unless our explanatory models descend to the individual level—a cardinal requirement for any intervention—their real-world applications will always be limited. Drawing on an analysis of the informational properties of the brain, here we argue that adequate individualisation needs models of far greater dimensionality than has been usual in the field. This necessity arises from the widely distributed causality of neural systems, a consequence of the fundamentally adaptive nature of their developmental and physiological mechanisms. We discuss how recent advances in high-performance computing, combined with collections of large-scale data, enable the high-dimensional modelling we argue is critical to successful translation, and urge its adoption if the ultimate goal of impact on the lives of patients is to be achieved.

## The question

Cognitive neuroscience is yet to produce applications of major clinical impact. If its relative immaturity is to blame, we need merely wait. But if its approach is fundamentally ill-suited, we could be left waiting forever. We must therefore consider how well the means of cognitive neuroscience support translational ends. Such consideration cannot be expected to emerge spontaneously from the field itself, for neuroscience evolves under the selective pressure of supposed understanding, not the collateral of mechanistic insight translation is widely perceived to be. Nor may we presume the obstacles to translation to be peculiar to each cognitive subfield and unlikely to be illuminated by a general analysis: it is possible they lie within the proximal, cardinal steps common to all of neuroscience. Here we examine this possibility, show it to be overwhelmingly likely, and outline how neuroscience must change if it is to deliver real-world patient impact.

## Translation and individualisation

Most societies give primacy to the individual person, imposing collective interests only with reluctance. This is especially true of healthcare, where the object of clinical action is archetypally the individual and the group only secondarily. To take a striking example, we could overnight revolutionize population outcomes in acute stroke by intervening with thrombolytic therapy at the kerbside, bypassing delays that hospital transfer for diagnostic computed tomographic scans inevitably introduce ( Wardlaw *et al*., 1997). But however compelling the population statistics, such a manoeuvre is rendered unconscionable by the resultant death or greater disability of the 10% of patients with primary intracerebral haemorrhage ( Qureshi *et al*., 2009). Even where the stakes are less sharply polarised, it remains difficult to implement any treatment whose individual benefit is only crudely probabilistic, for all interventions have a cost: both personal and financial. Moreover, since populations merely summarise effects on individuals, the greater the individual variation, the lesser the population-level impact. Both constitutively and politically, translational success or failure is thus critically dependent on our ability to individualise our interventions.

**Figure 1.**
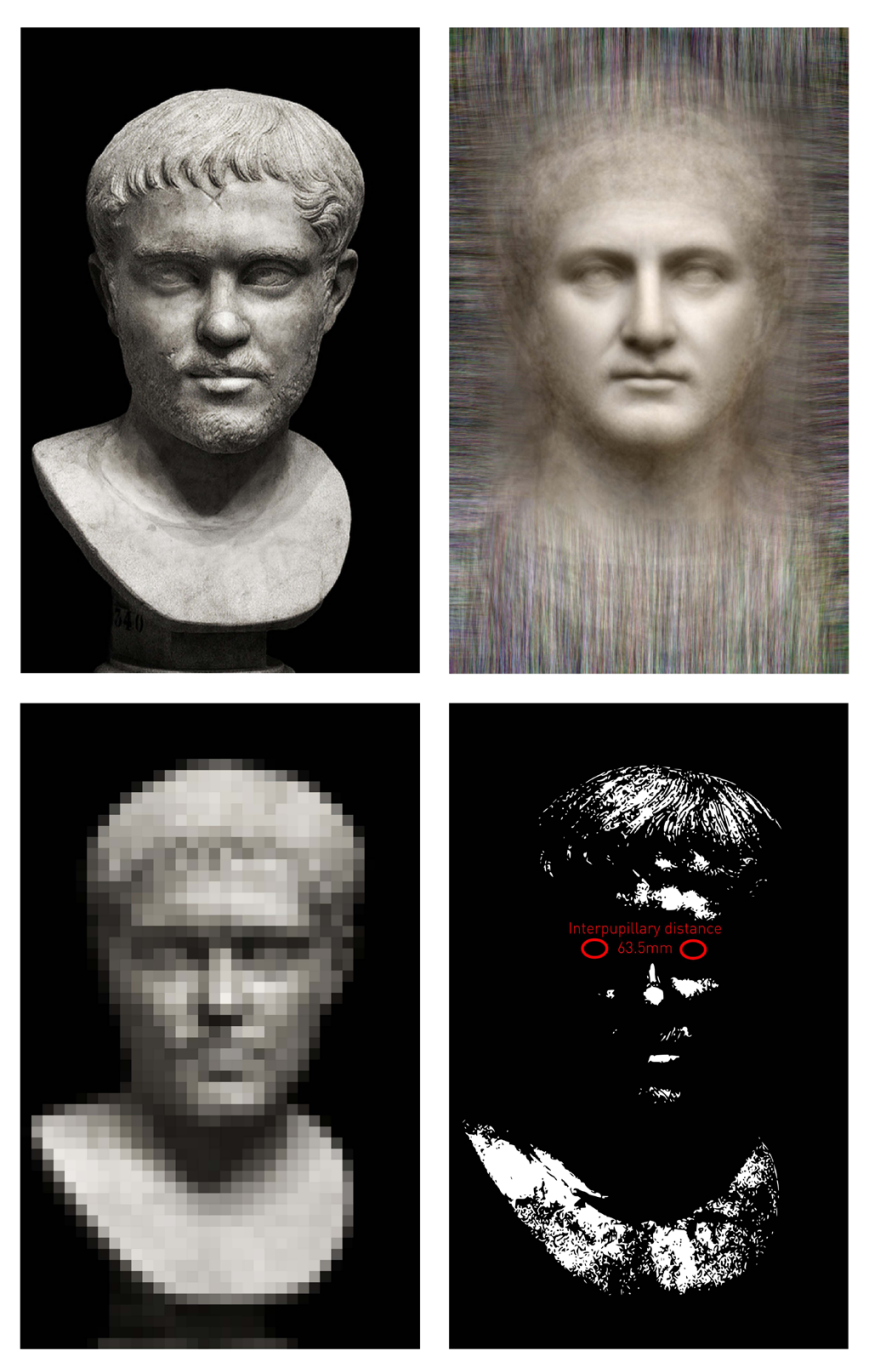
Dimensionality and individualisation. The face of the Roman Emperor Hostilian (top left) is poorly described by the canonical face of all Roman Emperors (top right), which is—by definition—not identical with any of the individual faces from which it is derived. Furthermore, the individuality of a face is better captured by a low-precision, high-dimensional parameterisation (bottom left), than it is by a high-precision, low-dimensional parameterisation such as the inter-pupillary distance (bottom right). The photograph of Hostilian is reproduced with the kind permission of Dr William Storage.

How is individuality determined? Consider by way of illustration that most personal part of the body, the face ( Figure 1). Though one feature may sometimes be uniquely idiosyncratic, to distinguish a face from another generally requires the conjunction of *many* features, even when all redundancy is eliminated. Such irreducibly high intrinsic dimensionality is conveniently captured by the notion of **minimum description length** ( Rissanen, 1978) – intuitively, the most compressed complete description of a system. This quantity sets a hard limit on the minimal complexity of any model that must distinguish one state or instance of a system from another to perform its task. No matter how clever the mathematics, a machine vision model tasked with (say) classifying the sex of a face will always perform badly when starved of input features because no small subset of features contains the necessary information; conversely, even a relatively unsophisticated model with sufficient capacity will perform well, given enough data ( Parkhi *et al*., 2015; Schroff *et al*., 2015; Zhou *et al*., 2015). It should come as no surprise that face coding in the primate brain takes a high-dimensional approach, deriving identity by projecting a multiplicity of features onto a compacted representational space ( Chang & Tsao, 2017). Now our concern is not individuation *simpliciter* but the individuation of causal mechanisms of predictive or prescriptive utility. For this we need a *causally constrained* extension of the concept of minimal description length: what we here term a **minimal causal field**. To see how this is specified requires a brief examination of biological causality.

## Neural causation

We have a natural intellectual predisposition to causal models with two cardinal features: economy and seriality ( Hacker, 2007). This is a consequence partly of reasoning by analogy and partly of practicability. The intelligible, mechanistically pellucid processes we observe in the non-organic world and exploit in the machines we build tend to have few parameters of causal significance, arranged sequentially. It seems natural to apply the same approach to biology, indeed inevitable, for a causal model with (say) a thousand parameters is intellectually intractable. When we insist on identifying *necessary* and *sufficient* links within a more or less serial chain, it is because no other option has been open to us.

**Figure 2.**
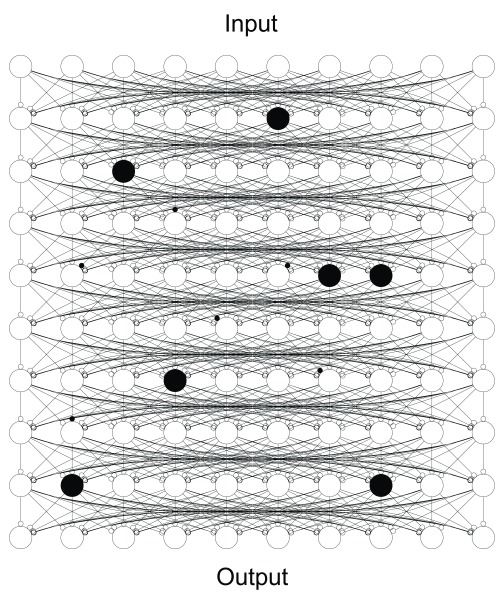
Causal fields. Distributed causality is elegantly illustrated by the behaviour of artificial neural networks **trained** to transform an input into an output by optimising the weights of a stack of fully connected nodes. Here the input-output transformation is causally dependent on the nodes and their connections, for it cannot occur without most of them. But when the network is large, its dependence on any limited subset of nodes will be low. This is not because there is a reserve of unused nodes, but because the causality of the system is constitutionally distributed. Inactivating (in black) a set of nodes (large circles) or their connections (small circles) will thus degrade performance broadly in proportion to their number and not necessarily their identity. Causality thus becomes irreducible to any simple specification of necessity and sufficiency. Instead, each node becomes an insufficient but necessary part of an unnecessary but sufficient set of factors: an INUS condition. An adequate description of the causality of the system as a whole then requires specification of the entire *causal field* of factors: no subset will do, and no strong ranking need exist between them. If the architecture of real neural networks makes such causality possible—and it certainly does—we need to be capable of modelling it. But this is more than just a theoretical possibility. It is striking that encouraging distributed causal architectures through dropping nodes or connections during training dramatically improves the performance of artificial neural networks. And, of course, real neural substrates often exhibit remarkable robustness to injury, a phenomenon conventionally construed as “reserve”, but since no part of the brain lies in wait, inactive, distributed causality is a more plausible explanation.

But whereas this notion of causation is adequate for understanding simple, serially organised systems, it does not scale with complexity. In complex systems, where a multiplicity of factors is *jointly* brought to bear on the outcome, each individual factor becomes an *i*nsufficient but *n*ecessary part of a set of factors that are *u*nnecessary but *s*ufficient for the result: an **INUS condition** ( Mackie, 1974). To give an adequately explanatory account it is necessary to specify a **causal field** of many such INUS conditional factors that interact in complex ways (see Figure 2).

Do neural systems require such a complexity of causal specification? Consider the far simpler behaviour of artificial neural networks, such as deep-learning architectures in which layers of laterally connected units are hierarchically arranged in an end-to-end error-minimising stack ( Goodfellow *et al*., 2016; LeCun *et al*., 2015). Taking the input-output transformation produced by such a network as its “function”, we can test the causal contribution of sets of network nodes by examining the functional consequences of deactivating them, essentially performing artificial neural network lesion-deficit mapping ( Adolphs, 2016; Rorden & Karnath, 2004). When a trained network is subjected to such **drop-out** ( Gal & Ghahramani, 2016; Le Cun *et al*., 1989; Srivastava *et al*., 2014; Wan *et al*., 2013), the degradation of any output is gradual, and often proportionate with the mass of deactivated nodes but varying with their identity in a complex manner that precludes the identification of a "critical" node, or even a clear ranking of the material contribution of individual nodes. Note the *architectural scale* of deactivation need not be eloquent either, for breaks in the hierarchy—observed in the brain and exploited in residual networks ( He *et al.*, 2016)—potentially allow entire layers to drop out without penalty ( Huang *et al.*, 2016). Causality is constitutionally *distributed* in a way any conventional description simply cannot capture; only a causal field specification will do ( Mackie, 1974).

The widespread use of drop-out in the deep-learning literature shows causally distributed architectures learn complex input-output transformations better than other systems examined to date ( Goodfellow *et al*., 2016). They are also more robust to damage, an important consideration for any biological system. However, there is no need to appeal to plausibility in our argument: to the extent that a node contributes to function it *must* be causally relevant. It is inconceivable that the observed complexity of real neural systems is merely epiphenomenal to a much simpler underlying causal organisation. In any event, since we cannot *assume* the minimal causal field is small, we need to consider how to model it when it is irreducibly large.

## Mapping causal fields

**Figure 3.**
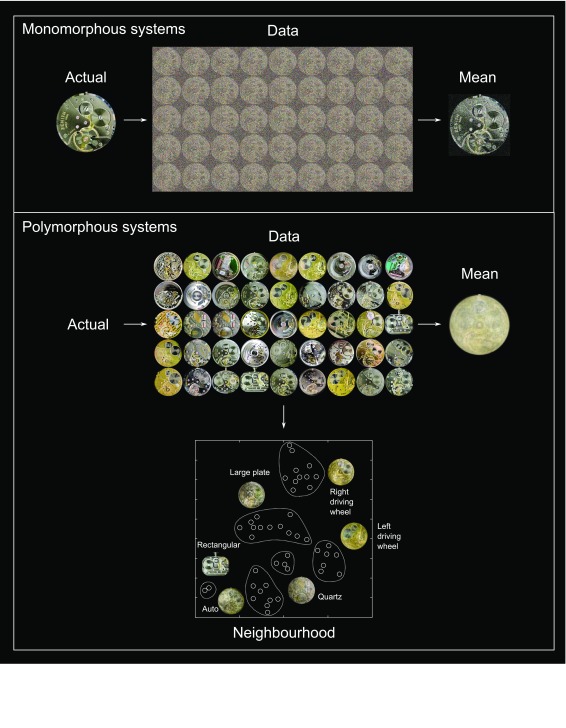
Monomorphous vs polymorphous systems. Where the fundamental architecture of a biological system is the same, our best guide will be the simple mean of the population, for each individual will differ from it randomly. Studying such monorphous systems is illustrated by adding random noise to an image of a specific watch mechanism, and then averaging across 45 noisy instances. The underlying architecture is thereby easily revealed. Where the solution in each individual differs locally, illustrated by taking a family of 45 different watch mechanisms of the same brand, the population mean is a very poor guide, for individual variability is no longer noise but the outcome of a plurality of comparably good solutions. We must instead define local regularities of organisation, here done by t stochastic neighbour embedding of the images into a two dimensional latent space, revealing characteristic features of each family of solutions. Given that neural systems are complex, stochastically initiated, and optimised by feedback, polymorphous architectures are likely to dominate, mandating a data-driven, neighbourhood-defining approach to modelling.

In seeking to understand the causality of any complex system, we must distinguish between causally relevant and incidental variation. Though rarely acknowledged, the approach to making such a distinction depends critically on a cardinal assumption about a system’s structure. If we assume the organisation of a particular brain network is fundamentally the same across people—i.e. it is **monomorphous**—individual variation may be treated as noise. The population mean will then be the best available guide to the fundamental mechanism and to its expression in individuals. This is an implicit assumption behind the vast majority of studies in cognitive neuroscience where a set of estimates, derived from a small group, are considered to reveal general truths about the brain. But if, at some causally critical level, the neural organisation is *not* the same across people—i.e. it is **polymorphous**—individual variation cannot be treated as noise and the population mean will be a poor guide, both to mechanism and individual behaviour (see Figure 3) ( Thiebaut de Schotten & Shallice, 2017). The distinction between monomorphous and polymorphous organisation is crucial because it radically alters the optimal inferential approach. We suggest the common assumption of a monomorphous architecture of the brain is unjustified—both empirically and theoretically—and must be discarded, for the following reasons.

## The genetic information gap

A neural architecture can be shared across individuals only as far as it is identically specified by the genome, the environment, and their interaction. The constitutive variability of the environment leaves the genome as the primary driver of inter-individual homology. Genomic information content is information theoretically limited by the number of base pairs and the range of nucleotide options at each locus. If we implausibly ( Rands *et al*., 2014) assume every locus is both functional and material to the operations of the brain, so that no section is redundant, we have only ~6 x 10 ^9^ bits of information, roughly the content of an old compact disc. Even if all this information is used to specify the minimal causal field of a human brain, leaving none for the rest of the body, we remain unable to meet even the most conservative estimates of the brain’s complexity. A commonly offered prenatal estimate, ~10 ^14^ bits, derived from the number of synapses in the brain ( Huttenlocher & Dabholkar, 1997; Tang *et al*., 2001), implausibly assumes a synapse can only encode one bit at any one time, and that neural connectivity is the only differentiator. This is equivalent to treating a neuron rather like a transistor in a modern computer-processing unit, distinguished from its neighbours only by the role assigned to it. In short, we are not faced with an information gap but more an information chasm. The conclusion is that a great deal of the functional architecture of the brain *cannot* be monomorphous, for the necessary information simply is not there.

## Creating polymorphous architectures

The brain cannot violate the laws of physics, so how can complexity arise from so relatively impoverished an initial specification? Theoretically the simplest approach is to inject *randomness* ( Matsuoka, 1992) at the outset of development, allowing a complex order to emerge downstream through *feedback learning*.

Such stochastic initiation is evident in normal neural development, where as many cells face an orchestrated death, at great structural and energetic cost to the organism, as survive into adulthood ( Lossi & Merighi, 2003). Seemingly playing a compound game of “Russian roulette cum musical chairs”, developing neurons are subjected to an environmentally dependent selection process, determined only once development is in play. The process is not fully specified in the genome, or else the redundant neurons would never be born. The biologically dominant prohibition of regeneration in the central nervous system, far from being mysterious, is necessary where the organising information emerges during development, and is therefore stored only in the final product itself.

Equally, the ubiquity of neural feedback learning is evident in the way recurrence is so densely woven into the neural fabric. One-way brain pathways are an exception, not the rule ( Bressler & Menon, 2010). It could not be otherwise, for learning—here neural learning—is the only way an order more complex than the initial genetic specifications could conceivably arise.

Now a stochastically-initiated, feedback-learning system, with multiple tuneable parameters, will inevitably have many *different* solutions for the *same* target input/output transformation. It is therefore bound to be polymorphous. Crucially, there need be no mechanism for regularising such solutions across individuals to impose a higher, species-level order, for no such order need exist, even if it could be imposed. An organism adapts its structure in response to errors only within its *own* input-output transformations, not those of others; biology does not do federated learning ( McMahan *et al*., 2016).

Though our concern is to define the bounds of biological possibility our models must be able to cover, it is natural to seek empirical evidence of biological plausibility. The only credible evidence can come from a system that has been comprehensively characterised. Since our claim is about under-estimating complexity, we may as well pick a simple one. Consider, the gut of the lobster, or rather the stomatogastric sub-circuit, meticulously studied for decades by Eve Marder. Though absurdly simple anatomically, with only 30 neurons, and physiologically, with only regular peristaltic oscillation, the relation between the two is not only complex, but also polymorphous in precisely the way described. The same functional physiology can be arrived at from different individual neuronal “settings”, both across time in the same animal and across different animals ( Marder & Bucher, 2007). We cannot presume that the rules of human functional brain organisation are any simpler.

## Modelling polymorphous systems

We must confront the functional complexity of neural organisation before us if translation from mechanisms of disease to rational treatments is to be possible. How do we *generate*, *estimate*, and *validate* polymorphous neural models of potentially incomprehensible complexity?

Before we descend into the details, let us sketch out a general approach. Although a polymorphous system may be so complex that no individual is like any other, it is reasonable to expect a set of similarities or “family resemblances” from which the properties of unseen individuals can be inferred. That the population mean is an inadequate guide does not imply the centroid of the *local neighbourhood* is not informative. Our task is to characterise the biological terrain, at a granularity the data themselves compel, so that we may describe each individual in terms of membership of a characteristic neighbourhood. Neither the terrain, nor the dimensionality of the space it occupies, are known, and must be determined empirically and computationally. In essence, we must move from models that *assume* a low-dimensional population mean is our best guide, to models that *discover* a set of high-dimensional neighbourhoods that best describe each individual.

### Model validation

Let us begin with the last step first: validation. It is conventional to take goodness-of-fit, qualified by some statistical measure, as evidence for the plausibility and utility of a model. But this is of little use where the field of possible models is both vast and sparsely sampled. That our model shows a degree of fit with the data means little if uncountable very different models fit just as well or better. The practice is kin with awarding oneself a gold medal after finishing a race blind to the rest of the field. Nor is limited model comparison acceptable, for differentiating between a handful of models tells us little about the sea of possibilities from which they are drawn.

Rather, we need to quantify the *individual* predictive power of a model, across time or across individuals, in relation to the future state of a system, or some outcome measure of interest. Such prediction is naturally framed in standard terms of sensitivity and specificity, derived from a comprehensive spread of data the model has not seen ( Dwork *et al*., 2015; Vapnik, 1998). A model with perfect predictive power cannot be improved upon, so its competitors may be reasonably dismissed. A model with imperfect predictive power is to be stratified by metrics, leaving as much or as little room for exploring others as its performance dictates.

Crucially, if a given model is powerfully predictive, none of the constituent features can be treated as noise, no matter how random they may appear when viewed in isolation. This approach does not implicate any individual constituent feature mechanistically, because functionally irrelevant incidentals (data and/or features) may drive prediction, like correlation. But it does imply no component feature leading to a good prediction can be safely ignored.

Of course, the richer the parameterisation of a model, the more susceptible it is to “overfitting” - the identification of coincidences of features in a dataset arising by chance with no predictive power beyond it ( Hawkins, 2004). But that is no more reason for avoiding such an approach than the possibility of being dazzled is reason for keeping one’s eyes permanently shut. It is in any event a practical, not a theoretical objection, addressable through the use of large-scale, fully inclusive datasets and high performance computing, as we discuss below.

### Model generation and estimation

To insist on *intuiting* a hypothesis as the first investigative step imposes a bias towards models couched in familiar concepts within a contemporary sphere of comfort. Where the hypothesis space is too large for our imaginations to traverse confidently, relying on intuition is not principled but hubristic. We need a formal hypothesis generation step, explicitly driven by exploratory analysis of data at sufficient scale and with adequate dimensional richness. The optimal scale and dimensionality will vary unknowably with any specific problem, but since both are likely to be very large, practical feasibility shall generally be the limit ( Ghahramani, 2015).

The manner of model generation constrains subsequent model estimation. If the former requires high dimensionality so will the latter. We cannot assume the underlying causal field to be sparse, or that its components will be linearly separable. In attempting to compress the dimensionality of models—explicitly through the use of a feature selection step, or implicitly through the use of sparsity-promoting inferential methods—we need to watch the impact on individual predictive power, assessed over a sufficiently diverse sample. Where a smooth decrement in prediction performance is seen with feature reduction, the underlying system is likely to be polymorphous, and aggressive feature selection is likely to be counter-productive. Equally, we cannot reliably rank input features taken in isolation on their marginal contribution to predictability, for this necessarily ignores their interactions ( Dramiński *et al*., 2008).

In short, models need to be complex enough to be tractable only with the highest capacity inferential architectures, such as the neurally inspired forms that have so rapidly grown to dominate the field of machine learning, notably in vision research. As in that case, this conclusion reveals two crucial problems, namely sensitivity to data scale and interpretability, both widely discussed in the literature (e.g. ( Bzdok & Yeo, 2017)). Rather than rehearse the familiar difficulties they present, here we draw attention to a few unexpected possibilities they reveal.

## The blessing of dimensionality

We have seen that complex, polymorphous systems require irreducibly many variables to achieve individually meaningful predictions. The resultant expansion of the parameter space under-determines models in proportion to the small scale of commonly available data. This familiar *curse* of dimensionality ( Vapnik, 1998) makes good solutions hard to find and even harder to generalize, for the risk of purely accidental fits increases with the number of parameters.

But we should recognise that dimensionality also carries a blessing. Consider the parameterisations of contrasting dimensionality shown in Figure 1. Such individualisation as our low-dimensional parameterisation may achieve—here the inter-ocular distance—will be strongly dependent on the precision of measurement, for everyone is differentiated along a single dimension. In contrast, with a high-dimensional parameterisation—such as a crudely pixelated rendition of the image—the precision of each individual variable is much less important, for the signal is conveyed in the covariance across variables. Crucially, since the structure of the underlying high-dimensional pattern is unlikely to resemble instrumental or other sources of noise, we can achieve *greater* individualisation with *lower* quality data. This is intuitively obvious in our ability to recognize faces from noisy, low-resolution images, robust not only to affine transforms of the data such as contrast, zoom, and skew, but also to fairly complex non-linear distortions.

The conventional resistance to using routinely acquired data on the grounds of noise and heterogeneity is only justified where the analysis is low dimensional. When measuring (say) total grey matter volume, it matters that one scanner will generate consistently greater estimates compared with another. But when extracting the high-dimensional variation of grey matter concentration across the brain, such effects will drop out as irrelevant affine shifts that leave the complex, individuating covariance patterns intact.

Perhaps the most important objection to high-dimensional modelling—the scale of the data required—is thus addressable through collections for another purpose, obtained outside a research environment. In the domain of structural brain imaging, the obvious source is clinical imaging ( Frackowiak & Markram, 2015). Since brain imaging is carried out to resolve diagnostic uncertainty towards normality almost as often as away from it, such data need not be restricted to the realm of pathology. Similarly, though smartphones may fall short of the precision of dedicated psychophysical devices, their ubiquity and critical mediating role in life enable the collection of rich, high-dimensional data on a vast scale ( Teki *et al*., 2016).

Of course, the correct balance between data size and data quality is an empirical question, to be settled case-by-case. But we cannot assume the former must be gated by the latter, and discount a high-dimensional approach simply because a conventional psychophysical laboratory cannot scale to thousands of participants. Rather, we must reconsider what we actually need to know, and what human activity may collaterally disclose it.

## Living with opacity

What use are high-dimensional models if they are too complex to understand? Where outcomes are highly variable, as is the norm in cognitive neurology, prediction is clinically invaluable, not simply because patients are consoled by accurate prognosis but because interventions need to be guided by their individually predicted responses. If a “black box” predictor is the best guide to a correct choice of treatment actuarially, it would be difficult to justify not following it *merely* because its operations cannot be paraphrased in intelligible prose.

Moreover, clinical interventions are already primarily driven by “black boxes” - the contents of our heads are a good example. To give a *reason* for acting is not to specify a cause or to imply an underlying causal model, it is kin with pointing to a latent variable. It is only rarely, where very simple biological systems are concerned, never in the brain, that we have a perspicuous, mechanistic explanatory model available. That a human expert can cite a reason for his actions does not make his decision-making less opaque than that of a synthetic counterpart.

If a system requires a complex model to describe it, then it *is* complex. Translational science needs to adjust to this emotionally, not hopelessly attempt to change it intellectually. A causal field so intricate it can only be specified as an artificial neural network with a million parameters *is* explanatory, even if its incomprehensibility makes us hesitate to use a word stronger than predictive. We can no more hope to understand the brain shackled to simple, linear models, than a literary critic could hope to understand Shakespeare applying the basic rules of grammar alone.

## Concluding remarks

Until a decade ago, the foregoing analysis would have been unbearably nihilistic, for we had neither the data nor the computational tools to realise the alternative it urges. The ground is still new and uncertain, yet to be proven capable of supporting the structure we argue it is imperative we begin to erect on it. But if we wish to move beyond discussions of tractability or feasibility into translatable action, we must confront the single most striking fact about the brain - its immense complexity.

The difficulties are all the greater for being distributed across many intellectual, technological, even political domains, reaching deep into the foundations of the very notion of biological understanding. A cognitive neuroscience recast in the form we propose will have more in common with meteorology than horology. If so, then it will be because the fundamental nature of the brain has compelled it, for what we urge here above all is to let the data, not our own brains, speak first. And if effective prediction supplants defective understanding as a result, those outside the field, whose lives cognitive neuroscience and cognitive neurology seek ultimately to serve, will appreciate the exchange.

## Data availability

No data is associated with this article.
