# Supplementary material for: Lost in translation
Source: F1000Res. 2019 Jan 2;7:620. Originally published 2018 May 21. [Version 2] doi: 10.12688/f1000research.15020.2 (PMC6107997; doi:10.12688/f1000research.15020.2)
Supplement: Peer review history

# Peer review: Referee response for version 1

**DOI:** 10.5256/f1000research.16353.r35675

**Published:** 2018-08-23

**Authors:** Tor D. Wager

**Affiliations:** 1 Department of Psychology and Neuroscience, University of Colorado Boulder, Boulder, CO, USA

In “Lost in Translation”, Nachev et al. provide a bold and thought-provoking piece on the past and future of cognitive neuroscience. Their thesis is that the classic inferential tools of neuropsychology, which also formed some of the foundational building blocks of cognitive neuroscience, are inadequate to model and understand the complexity of the brain. New paradigms are needed, and Nachev et al. offer a fresh, theoretical argument for why we should embrace new concepts of distributed causality, nonlinearity, and models that move beyond averaging over individuals to better capture inter-individual variation. The implication is that only by embracing such models can cognitive neuroscience, and perhaps neuroimaging in particular, develop models that are accurate enough to predict individual performance and clinical status - in short, to be translationally useful.

 Nachev et al.’s position has much to recommend it. One anchor point in their argument is that the brain processes that drive (cause) feeling and behavior are distributed across neurons and/or brain systems—and, if this is the case, then models and measures with coarse, distributed features (even if noisy) will outperform those with only a few, high-precision features. I resonate with this point. There is substantial evidence that the neural representations underlying multiple forms of cognition, emotion, and action are population codes distributed across large numbers of neurons and (in the case of fMRI) brain regions and systems (e.g., for review, see Kragel et al. 2018 ^1^).  In fMRI studies, distributed predictive models that include activity across regions and systems can dramatically outperform those based on even the best single brain regions (for recent reviews see, e.g., Arbabshirani et al. 2017 ^2^; Bzdok & Meyer-Lindenberg 2017 ^3^; Woo et al. 2017 ^4^; Kragel et al. 2018, Figure 3 and text).  

*Units of analysis*

 The essential question when it comes to brain systems is “What are the units of analysis”? The authors imply that translational failures come from a localisationist approach, and provide a thought-provoking theoretical argument for why the brain - at least with respect to cognition, feeling, and behavior - ought to be treated as a system with broadly distributed causality. I agree in part. Lesioning isolated nodes in a neural network typically does not catastrophically, or selectively, impair performance largely because the single node (or neuron) is not the relevant unit of analysis. But if one were to lesion a *layer*, particularly a layer dedicated to a particular function as in the structured, brain-inspired networks of O’Reilly et al. (Aisa et al. 2008 ^5^; O’Reilly et al. 2017 ^6^), the effects on network behavior would be profound. Recent advances in opto- and chemogenetics allow for the targeting and activation/inactivation of distributed sets of neurons that collectively represent particular cognitions and actions (e.g., Ramirez et al. 2013 ^7^), with strong effects on behavior. Likewise, when studying ecosystems, the loss of individual organisms selected at random has little effect on the behavior of the system as a whole; but loss of a species can have a profound effect. The species, but not the individual, can be characterized as having necessary and sufficient roles in the system’s behavior. Likewise, individual neurons are likely not necessary or sufficient for anything, but neural populations are. 

 A related point is Nachev et al.’s critique of averaging over individuals, which also hinges on the issue of which units of analysis are averaged over. Nachev et al.’s “watch” example is an interesting case study. They show that averaging over visual images of watch mechanisms does not elucidate the nature of watches or produce anything like any of the individual watches. But the problem is not averaging per se - it is knowing what to average over. Pixels in an image of the watch mechanisms are simply not the right unit of analysis, so averaging over them is meaningless. However, the average watch has 2 gear wheels, 2 hands, and one battery; averaging over or otherwise characterizing the distributions over these properties makes sense.

 Perhaps we will discover that brain voxels are not the right features to average over, either (and I suspect that they are not!). Cognitive neuroscience converges with machine learning in that a big part of the endeavor is, and has always been, discovering the units (or features) and level of analysis that confer maximal ability to understand the mind and predict future behavior. 

*Monomorphous and polymorphous*

 Another interesting contrast that Nachev et al. make is the distinction between monomorphous populations, whose individuals are identical, and polymorphous populations, whose individuals vary. Their central argument is that because human brains are polymorphous, we should not characterize them using averages across individuals; rather, we should focus on more individualized models. 

 Clearly, humans are a polymorphous bunch. But does this mean that population-level studies that characterize averages - or, alternatively, develop multivariate predictive models of behavior across individuals - are useless? Really, the brain is both monomorphous and polymorphous, at different levels of analysis. Identifying patterns of commonality does not mean that all variation is noise. If I were trying to describe to a space-faring alien what a car looks like, I would not assume that all cars are identical and the difference between a Tesla and a Toyota is “noise”. But neither would I assume that every car is completely different, which would preclude any sort of common description at all. The brain is similar in this respect. Virtually all of us have an occipital lobe, which contains a primary visual cortex. We have a primary motor cortex, a hippocampus, an inferior frontal junction, each of which plays consistent roles in behavior across individuals. In my lab’s work, we find that systems that track and predict the intensity of evoked pain experience are very similar across individuals - for example, the same brain pattern responds to painful events to some degree in 95% of participants (the 5% might well be largely noise; see Zunhammer et al., N = 603). But this does not mean that individual differences are unimportant! Rather, these baseline commonalities are a launching point for understanding the ‘variations on a theme’ that make individuals different from one another.  

*Lost and found*

 A final reflection: It is true that cognitive neuroscience has not developed many translational applications that are used clinically or commercially (e.g., Woo et al. 2017). But this does not necessarily imply that cognitive neuroscience has failed so far.  We should remember that the goal of cognitive neuroscience has been to understand the physiological representation of thought and behavior - one of the thorniest challenges in all of science, and a basic (not clinical) goal at that. Like many forms of basic science, the hope is that by better understanding how the mind works, without tying it immediately to any commercial venture or practical application, will yield new ways of thinking about the brain and mind, which in turn will inspire future applications that were previously unimagined. In my own lab’s work on pain and emotion, I have learned that the gaps between science and commercial application are not just about limitations in the science. Even if current cognitive neuroscience-based models could reliably diagnose mental and brain health conditions in individual people with perfect sensitivity and specificity, there would be gaps related to business development, marketing, public understanding and policy, economic cost/benefit ratios, equal access, insurance reimbursements, and more. I believe that in the past 2-3 years, cognitive and clinical neuroscience has succeeded in developing models of brain function that could be useful for characterizing dementia, depression, pain, autism, and more. Their clinical and commercial success will depend largely on what society wants to do with the science.

 This does not, of course, take away from the point that translation is a worthy and useful goal, both from a humanitarian (e.g., Gabrieli et al. 2015 ^8^) and scientific perspective. Not only can it advance clinical applications, translation also provides a concrete, objective yardstick against which to evaluate our understanding of the brain. Clearly, we have a great distance to go; the good news is that we're moving forward.

I have read this submission. I believe that I have an appropriate level of expertise to confirm that it is of an acceptable scientific standard.

## References

[1] KragelPAKobanLBarrettLFWagerTD: Representation, Pattern Information, and Brain Signatures: From Neurons to Neuroimaging. *Neuron*.2018;99(2) : 10.1016/j.neuron.2018.06.009 257-273 10.1016/j.neuron.2018.06.009 30048614PMC6296466

[2] ArbabshiraniMPlisSSuiJCalhounV: Single subject prediction of brain disorders in neuroimaging: Promises and pitfalls. *NeuroImage*.2017;145: 10.1016/j.neuroimage.2016.02.079 137-165 10.1016/j.neuroimage.2016.02.079 PMC503151627012503

[3] BzdokDYeoBTT: Inference in the age of big data: Future perspectives on neuroscience. *Neuroimage*.2017;155: 10.1016/j.neuroimage.2017.04.061 549-564 10.1016/j.neuroimage.2017.04.061 28456584

[4] WooCWChangLJLindquistMAWagerTD: Building better biomarkers: brain models in translational neuroimaging. *Nat Neurosci*.2017;20(3) : 10.1038/nn.4478 365-377 10.1038/nn.4478 28230847PMC5988350

[5] AisaBMingusBO'ReillyR: The emergent neural modeling system. *Neural Netw*.2008;21(8) : 10.1016/j.neunet.2008.06.016 1146-52 10.1016/j.neunet.2008.06.016 18684591

[6] O'ReillyRCWyatteDRRhorlichJ: Deep Predictive Learning: A Comprehensive Model of Three Visual Streams. *arXiv [q-bio.NC]*.2017;

[7] RamirezSLiuXLinPASuhJPignatelliMRedondoRLRyanTJTonegawaS: Creating a false memory in the hippocampus. *Science*.2013;341(6144) : 10.1126/science.1239073 387-91 10.1126/science.1239073 23888038

[8] GabrieliJDEGhoshSSWhitfield-GabrieliS: Prediction as a humanitarian and pragmatic contribution from human cognitive neuroscience. *Neuron*.2015;85(1) : 10.1016/j.neuron.2014.10.047 11-26 10.1016/j.neuron.2014.10.047 25569345PMC4287988

# Response

**Published:** 2018-12-27

**Authors:** Parashkev Nachev

**Affiliations:** UCL, UK

We are grateful to Professor Wager for his thoughtful commentary on our paper, and are pleased he finds sympathy with the broad thrust of our argument. Our revision clarifies the points he raises, and we provide a few further thoughts below.

*Units of analysis*

We agree there is no warrant for presuming a neural system must be intelligible at a neuroscientist's chosen pet scale of analysis. It is essential to explore a wide variety of scales, including those intelligible only through high-dimensional modelling, where—as we argue in detail in the paper—the critical organisation is mostly likely to be found. But it is also true that no *single* scale of analysis need be sufficient, for the organisation may be distributed across a *multiplicity* of them. Indeed, the kind of compact causal structure biology is likely to favour—for the information theoretic reasons we give—is constitutionally widely distributed. Of course, necessity and sufficiency is easily obtained at a whole brain level of analysis, but such causality is merely permissive rather than properly explanatory of how thought and behaviour really arise. Might there be some isolated "intermediate" level where causality is plain yet the underlying relations are revealed in sufficient detail? We doubt it, but the only way to tell is through the high-dimensional modelling approach we describe in the second half of the paper.

*Averaging*

It is a criterion both of general explanation—to the satisfaction of the scientist—and individual prediction—to the satisfaction of the clinician—that one can generalise from known instances to unknown ones. In both cases a model *learns* from one set of data (even if it is not called learning in the conventional statistical literature) and extrapolates to another. When we criticise averaging, our target is specifically low-dimensional models whose form is intuitively specified. In a high-dimensional, necessarily data-driven model, averaging occurs too, but across a *local neighbourhood*, not across an arbitrarily defined group as a whole. And that neighbourhood is determined by the modelling process itself, guided by out-of-sample predictive performance rather than model statistics. The point is illustrated by the clock mechanism example given in Figure 3.

*Translation*

We are clinicians, and for us the value of thought is measured by its utility in action. Others may, of course, adopt a different perspective. But consider what the ultimate criterion of fidelity must always be. A neuroscientist may claim his beautifully perspicuous theory of the function of some aspect of the brain is explanatory because it fits a model with half a dozen dimensions derived from fifty people. But what do we tell the patients—often the majority—the model does not fit? That they are *noise*? This would be an acceptable excuse *only* if the predictive power of more complex models remains poor, and the unmodelled variance is indeed plausibly noise. Once a model with better—and of course generalisable—predictive power is found, the claim is fatally undermined, for what was previously discarded as noise is now explained. That the resultant explanation might be complex need not be to its detriment: generalisable individual predictive performance always trumps perspicuity. The primary concern of the clinician ought to be the scientist's too.

# Peer review: Referee response for version 1

**DOI:** 10.5256/f1000research.16353.r34258

**Published:** 2018-06-19

**Authors:** Michel Thiebaut de Schotten

**Affiliations:** 1 INSERM (French Institute of Health and Medical Research) U 1127, CNRS (French National Center for Scientific Research), UMR 7225, ICM Institute for Brain and Spinal Cord, Pitié-Salpêtrière Hospital, Sorbonne University, Paris, France; 2 BCBlab, Sorbonne University, Paris, France

In their excellent and timely contribution Nachev, Rees and Frackowiak tackle current limitation in the models used to understand the brain functioning and the translation of this knowledge to the clinical practice. The text is engaging, and the message is clear. The authors did not limit their focus on the current problems but also provide clear new solutions and recommendations for future generations. 

 May I suggest the part on Dimensionality and individualisation to be linked up to a recent editorial entitled 'is a *single* brain model sufficient' (Thiebaut de Schotten and Shallice Cortex 2017) ^1^. I think this is appropriate but I leave it as optional for the authors. 

 Again, thank you for this elegant contribution soon to become a classic in the field.

I have read this submission. I believe that I have an appropriate level of expertise to confirm that it is of an acceptable scientific standard.

## References

[1] Thiebaut de SchottenMShalliceT: Identical, similar or different? Is a single brain model sufficient?. *Cortex*.2017;86: 10.1016/j.cortex.2016.12.002 172-175 10.1016/j.cortex.2016.12.002 28041614

# Response

**Published:** 2018-12-27

**Authors:** Parashkev Nachev

**Affiliations:** UCL, UK

We thank Professor Thiebaut de Schotten for his appreciative comments, and for the reference now cited in the revised version.
